# Screening for Cognitive Impairments in Primary Blepharospasm

**DOI:** 10.1371/journal.pone.0160867

**Published:** 2016-08-15

**Authors:** Jing Yang, Wei Song, Qianqian Wei, Ruwei Ou, Bei Cao, Wanglin Liu, Na Shao, Hui-Fang Shang

**Affiliations:** Department of Neurology, West China Hospital, Sichuan University, Chengdu, Sichuan, China; University of Florida, UNITED STATES

## Abstract

**Backgrounds:**

Studies have reported that non-motor symptoms are an important component of primary dystonia. However, evidence supporting cognitive impairment in primary dystonia is limited and contradictory.

**Methods:**

We applied the Chinese version of the Addenbrooke’s Cognitive Examination-Revised and the Mini-Mental State Examination (MMSE) to screen for cognitive impairment in patients with primary blepharospasm. In addition, we investigated the relationship between performance on the Addenbrooke’s Cognitive Examination-Revised and quality of life as measured by the Medical Outcomes Study 36-item Short-Form (SF36).

**Results:**

The study included 68 primary blepharospasm patients and 68 controls matched by age, sex and education. The prevalence of cognitive deficits was 22.0% and 32.3% in primary blepharospasm patients group, as measured by the MMSE and the Addenbrooke’s Cognitive Examination-Revised, respectively. Primary blepharospasm patents had a broad range of cognitive deficits, with the most frequently affected domains being visuospatial function (30.9%) and language (30.9%), followed by memory (27.9%), orientation/attention (26.4%) and verbal fluency (22.0%). Patients with cognitive deficits had lower total SF36 scores, especially in the subdomains of physical functioning, role-physical and social functioning, compared to those without cognitive deficits. Scores on the Addenbrooke’s Cognitive Examination-Revised were significantly correlated with both the SF36 scores and the scores on the subdomains of physical functioning and social functioning.

**Conclusions:**

Some patients with primary blepharospasm have cognitive deficits. Poor performance on the Addenbrooke’s Cognitive Examination-Revised is related to poorer quality of life.

## Introduction

Dystonia is a common heterogeneous movement disorder that is characterized by sustained or intermittent muscle contractions, which results in twisting movements and/or abnormal postures[1]. Dystonia can be classified according to its etiology and clinical characteristics, including age of onset, body distribution, temporal pattern and associated features[1]. Despite the prominent motor symptoms, clinical studies have revealed that patients with primary dystonia suffer from a wide range of non-motor symptoms, including the more commonly reported sensory and neuropsychiatric abnormalities and the less frequent cognitive abnormalities[2]. Both motor and non-motor symptoms have been shown to decrease quality of life (QoL) in patients with dystonia[3]. Although the etiology and pathophysiology of primary dystonia remain incompletely understood, dystonia is associated with basal ganglia dysfunction, and there is increasing evidence that the basal ganglia plays a role in both cognitive functions and motor control [4]. In addition, several neuroimaging studies have indicated that there are abnormalities in non-motor areas and the cortico-striatal-thalamo-cortical circuits in patients with dystonia[5]. Therefore, non-motor features, such as abnormalities in sensory processing, neuropsychiatric problems and cognitive impairments, are not surprising. [2, 6].

Although several studies have investigated cognitive abnormalities in primary dystonia, the results have been conflicting. Scott et al. studied a clinically heterogeneous group of fourteen primary dystonia patients using the Cambridge Neuropsychological Test Automated Battery, and found that patients with dystonia have a constellation of attentional-executive cognitive deficits compared to normal controls, whereas the speed of information processing, language, spatial, memory and general intellectual skills were well preserved[7]. Attention deficits were also reported in a study involving nine patients with primary cranial dystonia [8]. Conversely, another study of ten patients with primary dystonia, who differed in terms of body distribution, detected that patients with dystonia had significantly lower word fluency than the healthy controls, but no deficits in executive function or working memory[9]. Dias et al found that executive function, as assessed by the Frontal Assessment Battery, was not altered in primary blepharospasm (BSP) compared with hemifacial spasm[10].

Although the above evidence using different neuropsychological batteries shows contradictory alterations in cognitive functions in patients with primary dystonia, we should note that most of these studies are of small sample size and did not control for the type of dystonia, accompanying mood symptomatology or potential medication effects when assessing cognitive performance. The different sensitivities of the neuropsychological batteries adopted in these studies with different research focuses and the use of different controls groups may have also contributed to the inconsistent findings and hindered the cross-study comparisons.

A recent study found that a series of twenty BSP patients performed significantly worse on the Luria sequencing test, Purdue pegboard test, reciprocal coordination, tactile denomination, and reverse visuospatial span compared to the controls[11]. These findings suggested that BSP patients have a wide range of cognitive impairments, including impairments in planning complex movements, motor dexterity, visuospatial working memory and tactile object recognition. Given that lengthy neuropsychological batteries are often not feasible in routine practice or large-scale studies, there is a need to develop short cognitive tests that are simple to administer, assess a wide range of cognitive domains and are sensitive to frontal/executive and attentional deficits. The Addenbrooke’s Cognitive Examination-Revised (ACE-R)[12], which incorporates the widely used Mini–Mental State Examination (MMSE)[13], was developed as a multidimensional cognitive test. Different versions of the ACE-R have been validated in several countries and have been used in a number of neurodegenerative disorders to evaluate cognitive dysfunction and reflect disease progression [14–16]. The Chinese version of the ACE-R has been shown to be a reliable assessment tool to screen for mild cognitive impairment (MCI) and Alzheimer’s disease, with cut-off scores of 86 and 68, respectively[14].

Given the different descriptions of cognitive dysfunction in primary dystonia and the close relationship between basal ganglia dysfunction and cognitive disturbances, we aimed to investigate the spectrum and features of cognitive impairment in primary blepharospasm using the Chinese versions of both the ACE-R[14] and the MMSE. In addition, we explored the potential associations between cognitive function and clinical variables., Further, we investigated the effect of ACE-R scores on QoL using the Medical Outcomes Study 36-item Short-Form(SF36)[17].

## Patients and Methods

### Subjects

In total, 68 primary BSP patients were recruited from the Movement Disorder Center in the Neurology Department at West China Hospital of Sichuan University between December 2013 and May 2015. Known causes of secondary dystonia were excluded on the basis of medical and drug histories, neurological examination, laboratory investigation and abnormal findings on conventional MRI. In addition, all patients had no neurological abnormalities except BSP and had no family history of movement disorders. To guarantee that all patients could fully understand the cognitive tests and avoid large variations in education levels, patients with fewer than 8 years of education were excluded. The demographic features and clinical data, including the cognitive screening of both the patient and control groups, were collected by a neurologist during a face-to-face interview in a clinic room that was used for clinical evaluations. The severity of BSP was assessed using the Jankovic Rating Scale (JRS), which is a 4-point scale that includes both the severity and frequency of the involuntary orbicularis oculi muscle spasms [18]. QoL for BSP patients was evaluated using the SF36, which assesses physical and mental well-being in social and individual contexts[17]. Mood symptoms were evaluated using the Hamilton Anxiety Rating Scale (HAMA)[19] for anxiety and Hamilton Depression Rating Scale (HAMD; 24 items) for depression[20]. A group of 68 healthy controls with no history of neurological or psychiatric disorders and matched by age, sex and education level were chosen from the same region. All participants were right-handed. This study was approved by the Ethics Committee at West China Hospital of Sichuan University (S1 and S2 Files), and written informed consent was obtained from all participants.

### ACE-R administration

The Chinese version of the ACE-R takes approximately 15 minutes to administer and comprises five cognitive domains: attention/orientation (18 points), memory (26 points), fluency (14 points), language (26 points) and visuospatial function (16 points). The maximum score is 100 points, and a higher score represents better cognitive function. The ACE-R also encompasses the MMSE score (30 points), which was extracted and used as a comparison measure in this study. Cognitive deficit was defined as a total score less than 1.5 standard deviations of the controls’ mean on the ACE-R, or a total score of less than 27 on the MMSE by age and educational level[21].

### Statistical analysis

Statistical analysis was conducted using SPSS 17.0. The results of the continuous data are presented as the mean ± standard deviation. Comparisons of continuous variables between groups were conducted using Student’s t-tests when the variables met the normal distribution or Mann-Whitney tests when the variables did not meet the requirements for Student’s t-test. Chi-square tests were applied to compare categorical variables. Potential determinants of cognitive impairment (sex, education level, age of onset, disease duration, JRS-total score, HAMA score, HAMD score and use of drugs) were explored using binary logistic regression models. The relationship between the ACE-R and SF36 scores was assessed using a ranked sixth-order Pearson’s partial correlation analysis (for nonparametric data) with age, education level, disease duration, HAMD score, HAMA score and JRS-total score as covariates. To exclude the potential effects of pharmacological treatment on cognitive performance, data from patients without pharmacological treatment were subjected to a secondary analysis. All ACE-R analyses were performed by an independent rater who was not involved in the collection of the neuropsychological data.

## Results

A total of 68 patients and 68 healthy controls, matched for age (*p* = 0.494; Student’s t-test), gender (*p* = 0.569; Chi-square test) and education level (*p* = 0.239; Mann-Whitney tests; education level ranging from 9–16 years for the patients and 9–17 years for the controls), were included in the analyses. The demographic data of the patients are shown in Table 1. Eighteen patients were taking oral pharmacological treatments for BSP. The most commonly prescribed medication for those patients was anticholinergics (15/18), followed by clonazepam (11/18), antipsychotics (11/18), baclofen (10/18), anti-depressant (3/18) and anticonvulsants (2/18). Seven patients received only botulinum toxin injections. In addition, 11 patients had previously received oral medication for a short time but discontinued due to unsatisfactory efficacy. Furthermore, 32 patients were newly diagnosed and had not received any previous treatment.

**Table 1:** Clinical and cognitive features of the BSP patients based on the ACE-R scores.

Variables	All patients (n = 68)	Patients with ACE-R<80 (n = 22)	Patients with ACE-R≥80 (n = 46)	p-value
Sex (M/F)	18/50	1/21	17/29	0.007*
Age of onset (years)	49.20 (10.95)	52.39 (8.63)	47.67 (11.68)	0.096
Mean age (years)	52.31 (11.56)	55.32 (9.17)	50.87 (12.38)	0.139
Disease duration (years)	3.11 (2.98)	2.93 (2.09)	3.20 (3.34)	0.680
Education (years)	11.15 (2.59)	9.68 (1.49)	11.85 (2.72)	0.001*
MMSE scores	27.75 (2.14)	25.91 (2.41)	28.63 (1.29)	0.000*
ACE-R total scores	81.13 (11.67)	67.95 (10.31)	87.43 (5.26)	0.000*
Orientation/attention (max 18)	16.90 (1.49)	16.05 (2.06)	17.30 (0.89)	0.001*
Memory (Max 26)	22.47 (3.63)	19.77 (4.76)	23.76 (1.96)	0.000*
Verbal Fluency (Max14)	9.46 (2.46)	7.96 (2.43)	10.17 (2.15)	0.000*
Language (Max 26)	18.57 (4,86)	13.46 (3.76)	21.02 (3.09)	0.000*
Visuospatial (Max 16)	13.69 (3.47)	10.72 (4.68)	15.11 (1.18)	0.000*
JRS-severity	2.72 (0.97)	2.95 (0.72)	2.61 (1.06)	0.173
JRS-frequency	2.57 (1.06)	2.77 (1.02)	2.47 (1.09)	0.292
JRS-total	5.20 (1.99)	5.72 (1.61)	4.96 (2.13)	0.138
HAMD	9.24 (7.52)	11.77 (8.52)	8.02 (6.76)	0.079
HAMA	5.90 (5.36)	7.27 (5.81)	5.23 (5.06)	0.168
Physical Functioning (PF)	77.06 (21.04)	68.41 (22.17)	81.20 (17.88)	0.026*
Role-Physical (RP)	40.44 (40.53)	23.86 (34.05)	48.37 (41.30)	0.013*
Bodily Pain (BP)	79.83 (19.56)	76.63 (22.80)	81.37 (17.87)	0.398
General healthy (GH)	52.98 (21.75)	47.00 (22.83)	55.85 (20.86)	0.117
Vitality (VT)	64.19 (19.44)	58.41 (21.79)	66.96 (17.81)	0.118
Social Functioning (SF)	64.89 (23.58)	51.70 (22.92)	71.20 (21.38)	0.002*
Role-Emotional (RE)	53.92 (41.94)	48.48 (43.31)	56.52 (41.50)	0.472
Mental Health (MH)	58.00 (20.56)	55.27 (19.23)	59.30 (21.25)	0.454
SF-36	60.99 (17.40)	52.40 (16.39)	65.50 (16.50)	0.004*
Oral drugs (Y/N)	18/50	7/15	11/35	0.489

Notes: JRS, Jankovic Rating Scale; HAMA, Hamilton Anxiety Rating Scale; HAMD, Hamilton Depression Rating Scale.

The demographic data of the healthy controls and the cut-off values for the ACE-R total score and the different subdomains are presented in Table 2. Patients with BSP had significantly lower MMSE scores, ACE-R total scores and ACE-R subdomain scores compared to the controls (p<0.05; Mann-Whitney test). Based on the MMSE scores, 15 patients (22.0%) had cognitive impairment. The cut-off score for the ACE-R was set as “80” for cognitive impairment. This resulted in a prevalence of cognitive impairment in the BSP patients of 32.3%. Patients with a total score less than 80 had significantly lower scores in every subdomain. The most frequently impaired domains were the visuospatial (30.9%) and language (30.9%) domains, followed by the memory (27.9%), orientation/attention (26.4%) and verbal fluency (22.0%) domains. Patients with cognitive impairment had significantly lower levels of education and included a higher proportion of females (Table 1). Patients with cognitive impairments also had significantly lower SF36 scores, especially in the subdomains of physical functioning, role-physical and social functioning, compared to patients without cognitive impairments. There were no significant differences in the age of onset, age, disease duration, disease severity, HAMA score, HAMD score and the percentage of patients taking oral medications between the patients with and without cognitive impairments.

**Table 2:** Normative data of the healthy controls on the ACE-R (n = 68).

variables	Range	Mean (SD)	Cut-off score
Education	9–17	11.68 (2.75)	-
Sex (M/F)	21/47		-
Age	21.53–76.52	50.98 (10.99)	-
MMSE	27–30	28.85 (1.22)	27
HAMD	0–10	2.53(2.72)	-
ACE-R (Max 100)	78–100	89.28 (6.22)	79/80
Orientation/attention (max 18)	14–18	17.73 (0.59)	16/17
Memory (Max 26)	18–26	23.99 (1.79)	21/22
Verbal Fluency (Max14)	5–14	10.66 (1.91)	7/8
Language (Max 26)	12–26	21.88 (3.37)	16/17
Visuospatial (Max 16)	9–16	15.01 (1.14)	13/14

The logistic regression model indicated that lower educational level (OR: 0.673, 95%CI: 0.488–0.929, p = 0.016) was a potential independent determinant of cognitive impairment in patients with BSP.

Total ACE-R scores were positively correlated with total SF36 scores, and the SF36 subdomain scores of physical functioning, social functioning and mental health (Table 3).

**Table 3:** Relationship between the ACE-R and SF36 scores in BSP patients (n = 68).

		Attention/ Orientation	Memory	Fluency	Language	Visuospatial	ACE-R Total
Physical Functioning (PF)	*r*	0.170	0.148	0.139	0.390	0.300	0.388
	*p*	0.187	0.252	0.282	0.002*	0.018*	0.002*
Role-Physical (RP)	*r*	0.221	0.118	0.126	0.024	-0.090	0.091
	*p*	0.084	0.362	0.331	0.851	0.487	0.480
Bodily Pain (BP)	*r*	0.146	0.258	0.177	0.057	0.043	0207
	*p*	0.257	0.043*	0.168	0.659	0.739	0.106
Role-Emotional (RE)	*r*	0.101	0.004	0.077	-0.003	-0.007	0.036
	*p*	0.433	0.974	0.552	0.982	0.958	0.784
Mental Health (MH)	*r*	0.246	0.302	0.123	0.193	0.009	0.270
	*p*	0.054	0.017	0.342	0.132	0.945	0.034*
Social functioning (SF)	*r*	0.168	0.199	0.019	0.289	0.260	0.318
	*p*	0.193	0.121	0.881	0.023	0.041*	0.012*
Vitality (VT)	*r*	0.214	0.177	0.046	0.025	-0.018	0.115
	*p*	0.096	0.169	0.724	0.845	0.891	0.374
General healthy (GH)	*r*	-0.014	0.036	-0.053	-0.105	0.198	0.107
	*p*	0.915	0.783	0.683	0.419	0.123	0.406
SF-36 total	*r*	0.262	0.209	0.110	0.242	0.194	0.319
	*p*	0.039*	0.102	0.395	0.058	0.131	0.012*

Notes: Ranked sixth-order Pearson’s partial correlation coefficients with age, education level, disease duration, HAMD score, HAMA score and JRS-total score as covariates.

* indicates a significant correlation.

After excluding the data of 18 patients who took oral pharmacological medications, the pattern and prevalence of cognitive impairments in the BSP patients who did not take oral medications were similar (Table 4). The logistical regression model showed that lower educational level remained a potential independent determinant of cognitive impairment in the BSP patients who did not take medications (Table 5). The total ACE-R score was positively correlated with both the total SF36 score (r = 0.325, p = 0.033) and the SF36 subdomain scores of physical functioning (r = 0.311, p = 0.042) and mental health (r = 0.356, p = 0.019) in patients without oral pharmacological medications.

**Table 4:** Cognitive impairments in BSP patients without oral pharmacological medications (%) (n = 50).

Variables	Cut-off score	BSP (n, %)
Orientation/attention (Max 18)	16/17	12, 24.0%
Memory (Max 26)	21/22	13, 26.0%
Verbal Fluency (Max14)	7/8	13, 26.0%
Language (Max 26)	16/17	15, 30.0%
Visuospatial (Max 16)	13/14	18, 36.0%
ACE-R (Max 100)	79/80	16, 32.0%
MMSE	27	11, 22.0%

**Table 5:** Determinants of cognitive impairments based on the ACE-R cut-off score (<80) (Data were from those fifty patients not in receipt of oral medication.).

Clinical variables	Multivariate analyses	
	Odds ratio (95%CI)	p-value
Gender (female/male)	0.216 (0.019–2.394)	0.212
Education	0.678 (0.469–0.982)	0.040*
Age of onset	1.037 (0.955–1.126)	0.388
JRS-Total	1.062 (0.680–1.656)	0.792
Disease duration	1.013 (0.755–1.361)	0.930
HAMD	1.020 (0.861–1.209)	0.817
HAMA	1.072 (0.818–1.406)	0.613

Notes: JRS, Jankovic Rating Scale; HAMA, Hamilton Anxiety Rating Scale; HAMD, Hamilton Depression Rating Scale.

## Discussion

To our knowledge, this is the largest study to explore cognitive deficits in one type of primary focal dystonia and the impact of cognitive deficits on QoL. This study shows that BSP patients have a wide range of cognitive deficits when compared with a group of healthy controls matched by age, gender and education level. The multivariate analysis revealed that lower education level was a potential independent determinant of cognitive decline. Patients with cognitive deficits had poor QoL, especially in the domains of physical functioning, role-physical and social functioning.

In the current study, we found that BSP patients have cognitive deficits at a prevalence of 32.3% based on the ACE-R and 22.0% based on the MMSE. The average MMSE score (28.85 ± 1.22) in our patients is similar to that observed in patients with primary generalized dystonia (27.8 ± 2.3)[22] and tends to be higher than that observed in another cohort of patients with BSP (25.3 ± 3.3)[10]. The different prevalence of cognitive deficits found using the ACE-R and MMSE suggests that the ACE-R is more sensitive than the MMSE for the detection of cognitive impairments, which is consistent with the results of a previous meta-analysis[23].

Several cognitive impairment domains were detected in the current study. Attention deficit, which was detected using the ACE-R, indicates frontal lobe dysfunction and is supported by recent studies of patients with different types of dystonia, including primary generalized dystonia and focal dystonia[7, 8]. Together with the findings from other studies indicating the presence of sustained attention deficit and impairments in planning and attentional set shifting in patients with primary dystonia[8, 11], attention deficit may indicate the disruption of the dorsolateral-prefrontal loop, which involves the striatum, thalamus and prefrontal cortex. A previous neuroimaging study by our laboratory and other studies have shown that these regions are associated with altered functional activity and microstructural abnormalities [5, 24]. Deficits in visuospatial functioning have also been observed in patients with spasmodic torticollis, which may reflect dysfunction in striatal-frontal circuits [25]. Memory deficit was reported in primary cranial-cervical dystonia using other neuropsychological batteries [11, 26]. The deficit in verbal fluency found in our study is consistent with the findings in myoclonus dystonia [27] and another group of primary dystonia patients with different types of body distribution [9]. The verbal fluency domain of the ACE-R includes phonemic fluency, which is associated with frontal lobe function and executive function, and semantic fluency, which involves the frontal and temporal lobes [28]. Neuroimaging studies have revealed altered functional activity and glucose metabolism in the frontal and temporal lobes in patients with BSP, which may represent the neuroanatomical basis for deficits in verbal fluency[29, 30]. The cognitive deficits found in our study and in previous studies support the theory of broad cortical involvement with disruption occurring either within the frontal-subcortical loops, which involves the basal ganglia, or within other non-motor regions, including the cingulate cortex, occipital lobe, parietal lobe or temporal lobe[5, 24]. The reported differences in prevalence rates of impairment within multiple cognitive domains suggests that cognitive impairment in patients with BSP may be heterogeneous rather than a universal feature and partially explains the inconsistent findings of previous studies, which included small heterogeneous samples and focused on only some aspects of cognition.

There is a view that the subtle cognitive alterations in some studies may be related to the distracting effects of abnormal movements, evidenced by improvements in attention deficit after treatment with botulinum toxin in patients with cervical dystonia[8]. Although some of the patients in our study were not under treatment at the time of evaluation, we think the motor disability found in the dystonia patients did not affect their scores on the cognitive tests because the tests were conducted on the patients without limitations on their response or performance times. The absence of a significant association between movement severity in the BSP patients and cognitive deficit also supports this theory. Among the different oral medications for dystonia, anticholinergic therapy is frequently shown to impair cognitive performance[31]. However, despite the adverse effects on cognition, most of our patients under treatment for BSP still choose to take low-dose oral pharmacological treatments over botulinum toxin injections because of the high cost of botulinum toxin in China. It is important to note that the prevalence and pattern of cognitive deficits were similar in all patients, regardless of whether they were taking pharmacological medications. In addition, low education level was an independent determinant for cognitive deficits in both groups.

Depression is a common non-motor symptom in BSP, as evidenced by the significant difference in the mean HAMD scores between the patients with BSP and healthy controls. The mean HAMD score for the patients with BSP was 9.24, and the mean score increased to 11.77 in the patients with lower ACE-R scores, which indicates the presence of mild depression. Although impaired mood can impact cognitive test scores, no significant difference in the HAMD scores was found between the patients with and without cognitive decline, and the multivariate analysis found that the HAMD score was not the determining factor for cognitive impairment in patients with BSP.

In the current study, the multivariate analysis revealed that lower education level was a potential independent determinant of cognitive decline. This finding is consistent with the findings form normative studies of diverse populations, which confirmed a dependent relationship between cognition and demographic variables, such as education[32]. Studies from other neurological diseases, such as Parkinson’s disease patients with MCI, showed that education level significantly affected performance on the ACE-R[33]. Therefore, education level should be considered when using the ACE-R to detect cognitive impairment in BSP patients.

Little is known about the impact of cognitive performance on QoL in dystonia. In the current study, significant differences in both the SF36 scores and some of the SF36 subdomain scores were found between BSP patients with and without cognitive deficits, which indicates that cognitive deficits may influence various aspects of QoL, particularly those related to physical and social functioning. The association between cognitive impairment and QoL suggests that efforts to improve health care for dystonia patients should not only focus on management of motor symptoms but also consider modifying cognitive deficits that contribute to poor QoL.

This study has several limitations. First, this is not population-based study but involves patients recruited from a single health center. Second, we did not use comprehensive neuropsychological batteries to test cognitive performance in more detail. Third, the cut-off scores used in our study should not be used in other samples of patients with dystonia in different countries where normative education levels may differ or when using other translated versions of the ACE-R.

In conclusion, some BSP patients have cognitive deficits. Poor performance on the Addenbrooke’s Cognitive Examination-Revised is related to poorer QoL.

## Supporting Information

S1 File Letter of approval from our ethics committee. (JPG)

S2 File The translated letter of approval from our ethics committee. (DOC)

S3 File STROBE Statement- checklist of items that should be included in reports of observational studies. (DOCX)
