# Supplementary figures and images for: Screening for Cognitive Impairments in Primary Blepharospasm
Source: PLoS One. 2016 Aug 15;11(8):e0160867. doi: 10.1371/journal.pone.0160867 (PMC4985064; doi:10.1371/journal.pone.0160867)

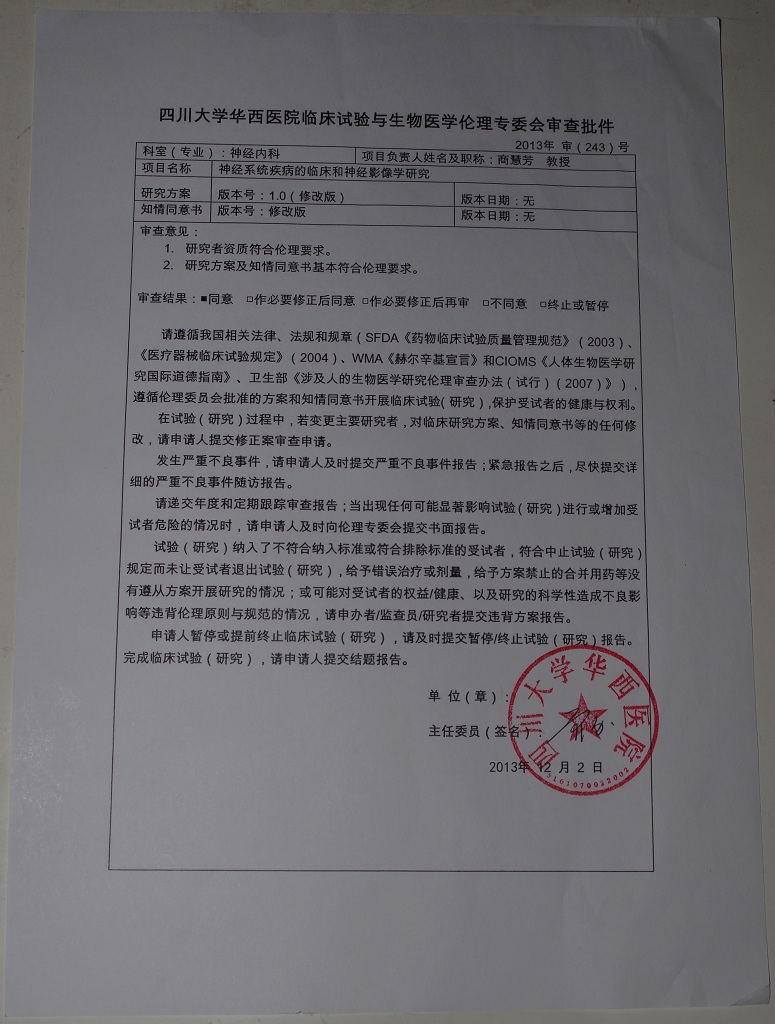

Supplement: S1 File — (JPG) [file pone.0160867.s001.jpg]
