# Supplementary material for: Screening for Cognitive Impairments in Primary Blepharospasm
Source: PLoS One. 2016 Aug 15;11(8):e0160867. doi: 10.1371/journal.pone.0160867 (PMC4985064; doi:10.1371/journal.pone.0160867)
Supplement: S2 File — (DOC) [file pone.0160867.s002.doc]

**Approval of West China Hospital of Sichuan University Clinical trials and Biomedical ethics committee**

**2013(243)**

| Department (major): neurology | | Project leader and title: Professor Huifang Shang | |
| --- | --- | --- | --- |
| Project name | The clinical and neuroimaging study of neurological diseases | | |
| Research project | Version :1.0 (modified) | | Version date: none |
| Informed consent form | Version: modified version | | Version date: none |
| Review comments:  1.the investigators qualify for ethical requirements  2.Research protocol and informed consent form meet the ethical requirements  Review result: ■ Agree  □ agree after necessary modification  □ review after necessary modification  □ disagree  □ terminate or suspend  Clinical trials( researches) should be conducted according to relevant laws and regulations: (SFDA《the Criterions for the Quality Control of Clinical Trial of Drugs》(2003), 《the clinical trials of the medical device regulation》(2004), WMA《Declaration of Helsinki》and CIOMS《International Ethical Guidelines for Biomedical Research Involving Human Subjects》, Ministry of Health《Ethical Review Procedures for Biomedical Research Involving Human Subjects (trial)》, as well as the approved protocol and informed consent form by the Ethics Committee to protect the health, rights and interests of subjects.  Review application of amendments should be submitted if there were any changes in the principal investigators or modification on the research protocol and informed consent form during the process of the research.  Report should be submitted as soon as possible once serious adverse event occurs, and detailed follow-up reports are required after the emergency report.  Annual and regular tracking review reports should be submitted; Written statements should be submitted to the Ethics Committee in time when research procedure is significantly affected or the risk of subjects is increased.  The applicant/ supervisor/ investigators should submit violation report once the following situation happens: 1) subjects who do not meet the inclusion criteria or those who meet the exclusion criteria were enrolled; 2) subjects who meet the discontinuation criterion were not suspended; 3) incorrect treatment or drug dosage were provided; 4) not complying with the research protocol such as administration of combined medication not allowed in the study; 4) violate ethical principles and standards such as having a adverse effect on subject’s rights and interests /health, or affect the scientific approach of the study.  Suspending or termination reports should be submitted if the applicant suspend or terminate the study in advance. A conclusion report should be submitted when the study is completed.  Organization (sealed)  Chairman (signature)    Dec. 2th 2013 | | | |
